# Novel Human Antibodies to Insulin Growth Factor 2 Receptor (IGF2R) for Radioimmunoimaging and Therapy of Canine and Human Osteosarcoma

**DOI:** 10.3390/cancers13092208

**Published:** 2021-05-04

**Authors:** Jaline Broqueza, Chandra B. Prabaharan, Samitha Andrahennadi, Kevin J. H. Allen, Ryan Dickinson, Valerie MacDonald-Dickinson, Ekaterina Dadachova, Maruti Uppalapati

**Affiliations:** 1 College of Pharmacy and Nutrition, University of Saskatchewan, Saskatoon, SK S7N 5E5, Canada; mlb457@mail.usask.ca (J.B.); sma936@mail.usask.ca (S.A.); kja782@mail.usask.ca (K.J.H.A.); 2 Department of Pathology and Laboratory Medicine, College of Medicine, University of Saskatchewan, Saskatoon, SK S7N 5E5, Canada; chp347@mail.usask.ca; 3 Department of Veterinary Pathology, Western College of Veterinary Medicine, University of Saskatchewan, Saskatoon, SK S7N 5B4, Canada; ryan.dickinson@usask.ca; 4 Department of Small Animal Clinical Sciences, Western College of Veterinary Medicine, University of Saskatchewan, Saskatoon, SK S7N 5B4, Canada; valerie.macdonald@usask.ca

**Keywords:** osteosarcoma, targeted radionuclide therapy, radioimmunotherapy, IGF2R, M6PR, antibody engineering, phage-display, one-health

## Abstract

**Simple Summary:**

Osteosarcoma (OS) is the most common type of bone cancer and mainly affects children, teens and young adults. The overall survival rate is ~67%, but patients with distant metastases have poor prognosis. Insulin growth factor 2 receptor (IGF2R) is a protein that has been shown to be expressed widely in human patient-derived OS cells and is a suitable for target for monoclonal antibody-based therapies. Given the similarities between canine and human OS, IGF2R is also overexpressed in canine OS. Towards the goal of one-health approach, we generated human antibodies that bind with similar affinities to IGF2R expressed in human, murine and canine tissues. We demonstrate tumor accumulation of radiolabeled antibodies in mice bearing human and canine patients derived tumors. Therefore, these antibodies show promise for development into the agents for radioimmunoimaging and radioimmunotherapy of OS in human and canine patients.

**Abstract:**

Etiological and genetic drivers of osteosarcoma (OS) are not well studied and vary from one tumor to another; making it challenging to pursue conventional targeted therapy. Recent studies have shown that cation independent mannose-6-phosphate/insulin-like growth factor-2 receptor (IGF2R) is consistently overexpressed in almost all of standard and patient-derived OS cell lines, making it an ideal therapeutic target for development of antibody-based drugs. Monoclonal antibodies, targeting IGF2R, can be conjugated with alpha- or beta-emitter radionuclides to deliver cytocidal doses of radiation to target IGF2R expression in OS. This approach known as radioimmunotherapy (RIT) can therefore be developed as a novel treatment for OS. In addition, OS is one of the common cancers in companion dogs and very closely resembles human OS in clinical presentation and molecular aberrations. In this study, we have developed human antibodies that cross-react with similar affinities to IGF2R proteins of human, canine and murine origin. We used naïve and synthetic antibody Fab-format phage display libraries to develop antibodies to a conserved region on IGF2R. The generated antibodies were radiolabeled and characterized in vitro and in vivo using human and canine OS patient-derived tumors in SCID mouse models. We demonstrate specific binding to IGF2R and tumor uptake in these models, as well as binding to tumor tissue of canine OS patients, making these antibodies suitable for further development of RIT for OS

## 1. Introduction

Osteosarcoma (OS) is the most common primary malignant bone tumor and the fifth most common primary malignancy among adolescents and young adults [1]. Unfortunately, overall survival has plateaued at approximately 70%, with no meaningful improvement realized in over 20 years [2,3]. Patients with metastases to the lungs and to the bone have an unfavorable prognosis with an overall survival of less than 40% and 20% respectively. Unlike some cancers, which share a common genetic signature, OS demonstrates sweeping genetic variability from one tumor to the next, marked by complex karyotypes and substantial aneuploidy [4]. This has made pursuing a conventional targeted treatment approach challenging and has led recent cooperative efforts to consider alternate approaches that involve commonalities such as metastatic patterns and tumor microenvironment [5]. Commonalities are becoming increasingly relevant given OS rarity, genetic variability and in some cases, resistance to conventional treatment strategies. Cation independent mannose-6-phosphate/insulin-like growth factor-2 receptor (IGF2R) is consistently overexpressed across multiple standard and patient-derived OS cell lines [6]. It had been shown previously that a single nucleotide polymorphism (SNP) within a haplotype block in IGF2R is associated with an increased risk of developing OS [7]. Taken together, IGF2R expression appears to be both essential to and associated with the development of OS, making it an ideal therapeutic target.

Targeted radionuclide therapy (TRT) involves the use of a radiopharmaceutical drug that specifically targets cancer cells. A radiopharmaceutical drug typically consists of an alpha- or beta- particle emitting radionuclide attached to a cancer cell targeting moiety. The ability to deliver cytotoxic radiation to tumor site with high precision via TRT avoids many adverse effects of external beam radiation therapy (EBRT). The promise of TRT is exemplified by the regulatory approvals of Xofigo (Radium-223 dichloride) for treatment of bone metastases of prostate cancer and Lutathera (Lutetium-177-Dotatate) for treatment of somatostatin receptor-positive gastroenteropancreatic neuroendocrine tumors (GEP-NETs). Radioimmunotherapy (RIT) is a form of TRT wherein the cancer targeting moiety is a monoclonal antibody that binds to antigens present selectively on cancer cells [8,9]. Zevalin, a radioconjugate of beta-emitter yttrium-90 to monoclonal antibody targeting CD20 (Ibitumomab), has been approved for treatment of refractory and recurrent non-Hodgkin’s lymphoma [10]. RIT has also been the subject of several clinical studies with promising results [9]. RIT is not affected by tumor resistance mechanisms and changes to the tumor microenvironment. Moreover, RIT enables systemic administration wherein the antibody mediated specific uptake in tumor minimizes the exposure of normal tissues, thereby enhancing the therapeutic window.

Our previous work demonstrated the preferential tumor localization of the radiolabeled monoclonal antibodies (mAbs) to human IGF2R (mAb MEM-238) and to human and murine IGF2R (mAb 2G11) in OS xenografts and patients derived xenografts (PDX) in mice in comparison with the control mAbs. Treatment of OS tumors using 188Rhenium (^188^Re) and 177Lutetium (^177^Lu)-labeled IGF2R-specific mAbs MEM-238 and 2G11 resulted in tumor growth inhibition and possibly regression while being safe to normal organs. Importantly, this tumor regression when comparison to multiple controls was achieved using a single administration and non-optimized dosing [11,12]. In addition, use of immunohistochemistry (IHC) using mAb 2G11 demonstrated positive binding to neoplastic osteoblasts on two randomly selected canine OS tumors [12].

To translate this novel approach into the clinic, two problems must be solved. First, generation of human monoclonal antibodies (mAbs) specific to IGF2R would prevent neutralization by immune system and allow multiple dosing of patients to achieve a cure. Second, demonstrating efficacy and safety of RIT with these mAbs in an animal model of compelling translational significance. Naturally occurring cancers in companion animals are a great resource, as shown by the remarkable growth of comparative oncology over the last 30 years. Cancer is a leading cause of death in companion animals. Furthermore, pet owners are seeking advanced and novel therapies for their pets. Living in the same environments, human and pets often suffer from the same types of cancer which show similar behavior, and in some species, expresses the same antigens [13]. Canine OS has comparable clinical presentations and molecular aberrations to human OS. Annually 10,000 new canine OS cases are diagnosed in the United States alone. Ninety percent of canine patients will die from metastasis within one year of diagnosis. As canine and human OS share certain antigens, this allows for the potential to target said antigens with the same mAbs [14,15], including mAbs to human cation independent mannose-6-phosphate receptor, which has been found to also bind to the canine counterpart [16]. In this study we describe development of novel human pan-antibodies to human, canine and murine IGF2R and their in vitro and in vivo evaluation as potential agents for radioimmunoimaging and RIT of canine and human OS.

## 2. Materials and Methods

### 2.1. Construction of Synthetic and Naïve Antibody Fab Phage-Display Libraries

A custom phagemid based on previously published pHP153 [17] was used for cloning and display of antibody variants in Fab format. This phagemid is partly derived from pBR322 and drives expression of antibody variants as a fusion to truncated gene 3 protein (C-terminal domain) under phoA promoter.

**Figure 1:**
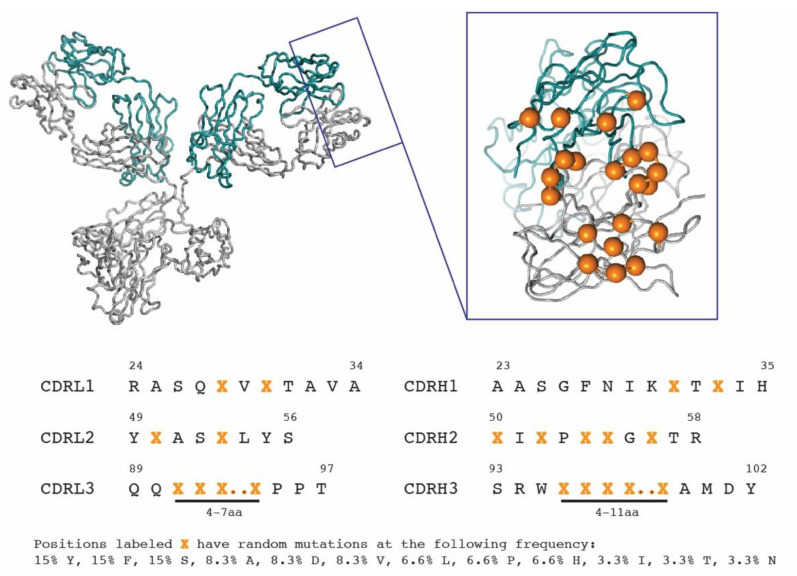
Design of synthetic antibody library based on 4D5-8 framework (Trastuzumab). Solvent exposed positions in CDR loops (orange) were selected for random mutagenesis to generate new binding interfaces.

For the synthetic library construction, humanized Her2 clone 4D5-8 (Trastuzumab) [18] framework was cloned in pHP153 vector and was used as a template for mutagenesis. The design of synthetic library is shown in Figure 1. Based on trastuzumab crystal structure (pdb 1N8Z), solvent exposed positions in complimentary determining regions (CDRs) were selected for mutagenesis. CDRL3 length was allowed to vary between 9–12 amino acids and CDRH3 was allowed to vary between 11–18 amino acids as described in Figure 1. All randomized positions were mutated with a custom (N1) HT codon, where N1 is a mix of 10%A, 20%C, 25%G and 45%T. The library was constructed using previously described methods using site-directed mutagenesis [17,19]. The DNA sequence of the 4D5-8 Fab template (VH and VL regions) and mutagenic oligonucleotides used for library construction are provided in Supplementary Methods.

For naïve library construction, a set of primary PCR primers described in Table S1, were used to amplify genes encoding antibody variable regions using cDNA derived from pooled peripheral blood monocytes of human blood donors (purchased from Takara Bio USA, Mountain View, CA, USA) according to previously described protocols [20]. A set of secondary PCR primers with vector specific overhangs (Table S2) were used to amplify VH and VL regions which were then spliced with intergenic constant region (containing CH1 and IRES sequences) using splice-overlap-extension PCR. The final PCR product was cloned into the phagemid using restriction digest with enzymes NsiI and NheI and ligation. The ligated DNA was then electroporated into *E.coli* SS320 pre-infected with M13K07 helper phage for phage library construction as described previously [19]. Primer sequences and library construction process is described in Supplementary Materials.

### 2.2. Production of Recombinant Human, Murine and Canine IGF2R Fragments

Gene fragments encoding the domains 11–13 of human IGF2R (aa 1511-1989, Uniprot P11717), murine IGF2R (aa 1504-1982, Uniprot Q07113) and canine IGF2R (aa 1515-1993, Uniprot B1H0W0) were generated using gene synthesis. These sequences (Figure S1) were cloned into pFUSE-hIgG1-Fc2 vector (Invivogen, San Diego, CA, USA) for generation of soluble Fc fusion proteins. Recombinant proteins were expressed in Expi293F cells (Invitrogen) and purified using MabSelectSure resin (GE Healthcare, Chicago, IL, USA), using manufacturer recommended protocols.

### 2.3. Phage Library Panning and ELISA

We used a modified approach to select for antibody variants that recognize IGF2R fragments from different species. The selection and ELISA methodology is essentially the same as described previously [19], with the modification that antigens were swapped every round. Briefly, recombinant IGF2R (positive selection) and Fc control (negative selection) proteins were coated by direct adsorption on NUNC Maxisorp 96-well plates at 5 µg/mL concentration in PBS. Phage library was pre-cleared for Fc binders by incubating on the negative selection plate for 1 h at room temperature. The library was then transferred to IGF2R protein coated plates for binding selection for 2 h at room temperature. The plate was washed 8 times with PT Buffer (1× PBS + 0.05% Tween20). Bound phage was eluted and amplified overnight in *E. coli* OmniMax T1R. We used human IGF2R fragment for Round 1 of selection, murine for Round 2 and canine for Round 3. Following 3 rounds of selection, 48 clones were analyzed from naïve library selection pool. For synthetic library, and additional round of selection was performed on human IGF2R fragment. 48 clones from synthetic library selection Round 4 pool were analyzed using ELISA using previously published protocols [19].

### 2.4. Production of Fab Fragments in Bacteria

The coding sequences for the VL and VH regions of selected Fabs were cloned into a custom expression vector with protein expression driven by a *ptac* promoter, with a C-terminal 6xHis tag at the end of CH1 domain on heavy chain. Fab proteins were expressed in *E. coli.* BL21 Codon Plus cells (Agilent, Santa Clara, CA, USA). Actively growing cells (O.D. 0.8) were induced using 0.4 mM IPTG and incubated at 24 °C for 12 h. Cells were lysed and the fab proteins were purified from clarified lysate using Ni-NTA Sepharose resin (GE Healthcare) using manufacturer recommended protocols.

### 2.5. Production of Full-Length IgG in Human Cells

For production of IgG version of lead antibodies, the VL and VH coding sequences were cloned into pFUSE2ss-CLIg-hK vector and pFUSE2ss-CHIg-hG1 vectors respectively (Invivogen). These clones were expressed in Expi293F cells using previously described protocols [21] and purified using MabSelectSure (GE Healthcare) affinity resin using manufacturer recommended protocols. SDS-PAGE were used to analyze the purity of the proteins.

### 2.6. ELISA of Purified IgG Binding to Recombinant IGF2R

Recombinant human, murine and canine IGF2R was coated at 3 µg/mL (in PBS) with 50 µL (0.15 µg) added to each well of NUNC Maxisorp 96-well plate. The wells were blocked with 100 µL PB Buffer (1× PBS + 0.2 mg/mL BSA) and left for one hour at room temperature. The plate was washed with PT buffer (1× PBS+ 0.05% Tween20) twice. Purified IgGs (IF1 and IF3) were added to the corresponding wells at concentrations ranging from 3 to 300 nM and incubated for 1 h at room temperature while shaking. The plate was washed 4 times with PT Buffer. Goat Anti-Human Kappa (K) Light Chain Antibody HRP (Invitrogen) secondary antibody was diluted (1:5000) in PBT buffer (1× PBS, 0.2 mg/mL BSA + 0.05% Tween20) and 50 µL was added to each well. The plate was incubated for 45 min at room temperature while shaking. The plate was washed with PT buffer four times and developed with 45 µL TMB subsrate (Seracare, Milford, MA, USA) for ~5 min. A total of 45 µL 1 M Phosphoric acid was added to each well to terminate reaction and the absorbance was read at 450 nm.

### 2.7. Reagents and Antibodies

The 2G11 mAb was obtained from ThermoFisher (Vancouver, BC, Canada) and was used as a positive control in flow cytometry experiments. Human mAb Palivizumab (IgG1) against respiratory syncytial virus (RSV) was acquired from MedImmune and was used as an isotype-matching negative control. SG-iTLC strips (Silica gel instant thin layer chromatography) were acquired from Agilent (Mississauga, ON, Canada). (R)-2-Amino-3-(4-isothiocyanatophenyl)propyl]-trans-(S,S)-cyclohexane-1,2-diamine-pentaacetic acid (CHXA’’) bifunctional chelating agent was purchased from Macrocyclics (Dallas, TX, USA). ^111^In was obtained from Nordion (Ottawa, ON, Canada); and ^225^Ac—from Oak Ridge National Laboratory (Oak Ridge, TN, USA).

### 2.8. Cell Lines

Human osteosarcoma cell line 143B and IGF2R negative murine cell line K7M2 were obtained from American Type Culture Collection (ATCC, Manassas, VA, USA). OS-33, a well characterized patient-derived osteosarcoma cells line, was a kind gift from Dr. R. Gorlick (MD Anderson Cancer Center, Houston, TX, USA). McKinley and Gracie canine patient derived osteosarcoma cell lines were a kind gift from Dr. Doug Thamm’s lab at Colorado State University School of Veterinary Medicine. The 143B cells were cultured in Eagle’s Minimum Essential medium. OS33 cells were cultured in Dulbecco’s Modified Eagle Medium and Gracie and McKinley cells were cultured in RPMI-1640 with HEPES. Media were supplemented with 10% FBS, sodium pyruvate, non-essential amino acids, and 100 U penicillin/0.1 mg/mL streptomycin.

### 2.9. Flow Cytometry

A 96-well plate consisting of 300,000 cells in each well were incubated with the different concentrations of the primary antibody (either 2G11, IF1, IF3, or RSV) ranging from 3 nM to 300 nM for 30 min. The RSV antibody (palivizumab) was used as a negative control. Cells were then washed twice with FACS Buffer (1× PBS + 0.5% BSA or 2% serum + 0.02% azide). The secondary antibody used for the IF1, IF3 and RSV antibodies was Goat anti-Human IgG Fc (PE) (eBioscience, cat. #12-4998-82). Goat Anti-Mouse IgG2a H&L (PE) from Abcam (ab74490) was used as a secondary for the 2G11 antibody. Secondary antibody was added and incubated for another 30 min. Cells were then washed again three times with FACS buffer before reading the plate in the CytoFlex machine.

### 2.10. Animal Models

Healthy six–eight-week-old SCID (CB17/Icr-*Prkdc^scid^*/IcrIcoCrl) female mice obtained from Charles River Laboratories (Wilmington, MA, USA) were used for the biodistribution experiments. For tumor induction the mice were anesthetized with isoflurane and injected subcutaneously with 4 × 10^6^ 143B, OS33, Gracie or McKinley cells into the right flank. Mice were monitored for tumor development, and imaging procedures were initiated when the tumors reached 5–7 mm in diameter.

### 2.11. Conjugation of Bifunctional Chelating Agent CHXA” and Radiolabeling of mAbs

One milligram each of IF1 and IF3 antibodies were conjugated with 2.5× or 10× excess CHXA” using previously described protocols [12]. The mAbs were radiolabeled with ^111^In and used in biodistribution and imaging experiments as described in [12]. IGF2R-Fab1 was conjugated with 10 M excess p-SCN-Bn-DOTA as in [12] and radiolabeled with ^225^Ac for in vitro cell killing as in [22]. Briefly, ^225^Ac chloride was diluted with 0.15 M NH_4_ acetate buffer and added to the Fab1-DOTA conjugate in the same buffer in a volume of ~35 μL. The reaction mixture was kept for 60 min at 37 °C, then quenched by the addition of 3 μL of 0.05 M EDTA solution to bind any free ^225^Ac. The radiolabeling percentage was measured by silica gel instant thin layer chromatography (SG-iTLC) with 0.15 M NH_4_ acetate buffer as the eluent. SG-iTLCs were cut in half and read on a Perkin Elmer 2470 Automatic Gamma Counter (top containing unlabeled ^111^In or ^225^Ac, bottom containing antibody labeled with ^111^In or ^225^Ac). For radiolabeling with ^111^In the counting of iTLC were counted immediately. For ^225^Ac radiolabeling iTLC were read 24 h after developing to allow for secular equilibrium to be reached. Yields were typically greater than 98% and required no further purification.

The radiolabeled antibodies were also analyzed by high performance liquid chromatography (HPLC). The Waters system used for analysis was equipped with UV detector, the flow through Bioscan radiation detector and a Toso Haas size exclusion column which was eluted with PBS at 1 mL/min.

### 2.12. In Vitro Cell Killing

The specificity of IGF2R-Fab1 fragment for IGF2R was tested in cell killing assay. One-hundred-thousand IGF2R positive 143B cells and IGF2R negative K7M2 OS cells in PBS were placed into the wells of the 96-well plate and treated with 0–500 nCi of ^225^Ac-labeled IGF2R-Fab1 for 1 h at 37 °C. As control the cells were also treated with 100 or 500 nCi of ^225^Ac-DOTA complex. After the incubation the cells were washed, placed in cell growth media and analyzed for survival by blue dye exclusion assay 72 h post treatment.

### 2.13. Biodistribution of ^111^In-Labeled IF1 and IF3 in Healthy Mice

Healthy mice were randomized and intraperitoneal (IP) injected with 30 μCi of either ^111^In-IF1, or ^111^In-IF3 prepared with 2.5 or 10 initial molar ratios of CHXA” to the protein. At 24 and 72 h post injection mice were sacrificed from each group. Once sacrificed the blood, spleen and right femur were collected, weighed, and counted in a gamma counter (Perkin Elmer, Waltham, MA, USA). The percent of injected dose per gram (%ID/g) for each sample was calculated.

### 2.14. microSPECT/CT Imaging of ^111^In-Labeled IF3 in Human and Canine Tumors in Mice

microSPECT/CT (micro single photon emission computer tomography/computer tomography) images were collected on a MILabs VECTor^4^ (Utrecht The Netherlands) microSPECT/CT scanner and processed using the comprehensive image analysis software package PMOD (version 3.9, PMOD Technologies, Inc, Zürich, Switzerland). Imaging studies were conducted using 200 μCi ^111^In-IF3. Tumor-bearing mice were administered ^111^In-IF3 via IV injection and imaged in the prone position at 24 and 48 h post injection. SPECT data were collected for 20 min using an Extra Ultra High Sensitivity Mouse (XUHS-M) collimator for 20–350 keV range using spiral trajectories. All SPECT images were reconstructed using both 245 keV and 171 keV ^111^In gamma emissions on a 0.4 mm voxel grid with MILabs reconstruction software.

### 2.15. Immunohistochemistry of Canine OS Tumors

Immunohistochemical detection of IGF2R in canine OS tumors was performed using an automated staining platform (Autostainer Plus, Dako Canada Inc., Mississauga, ON, Canada). Endogenous peroxidase activity was quenched using 3% hydrogen peroxide in methanol. Heat-induced epitope retrieval was performed in a Tris/EDTA pH 9 buffer for 20 min. The tissue was incubated with 1:25 dilution of IF1 mAb overnight at 4 °C. Bound primary antibody was detected using an HRP-labelled polymer detection reagent (EnVision+ System, Dako Canada Inc., Mississauga, ON, Canada) with 3,3′-diaminobenzidine tetrahydrochloride (DAB) (Dako Canada Inc., Mississauga, ON, Canada) as the chromogen and a hematoxylin counterstain. Pavilizumab was used instead of the primary antibody as the isotype negative control.

## 3. Results

### 3.1. Generation of Cross-Reactive Antibodies to Human, Murine and Canine IGF2R

**Figure 2:**
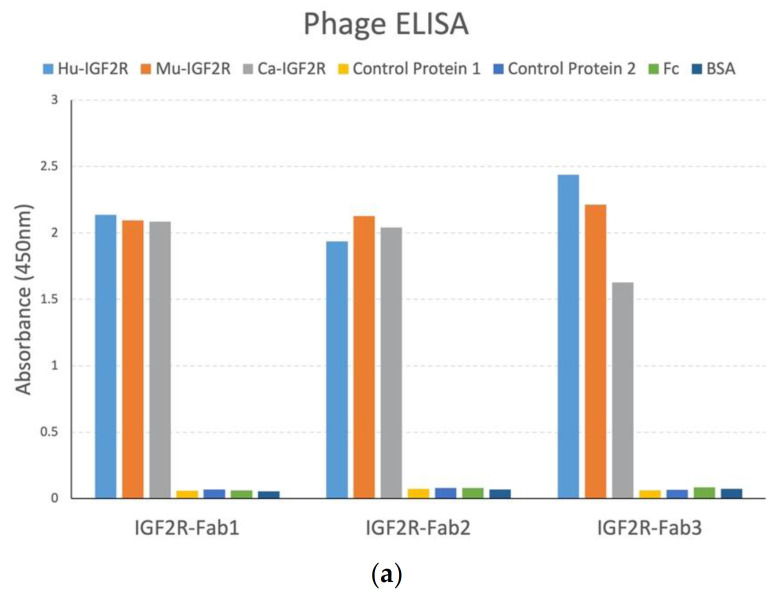

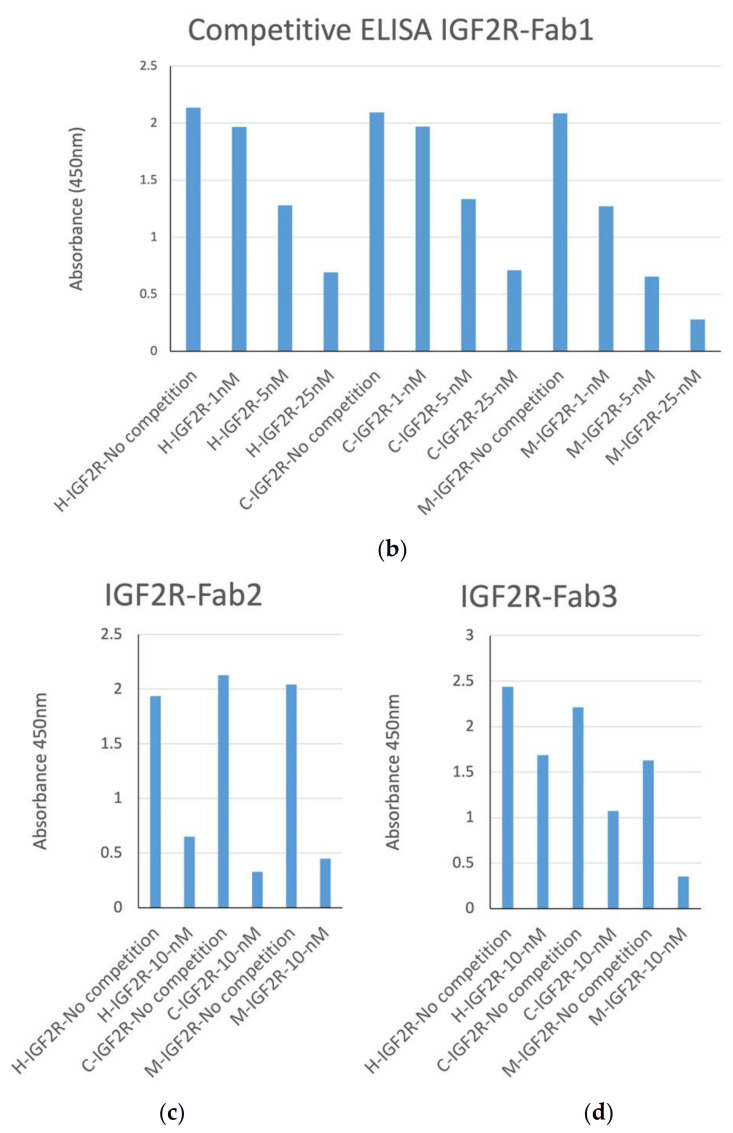
Fab-phage binding and competitive ELISA. (**a**) Selected antibodies bind specifically to human, canine and murine IGF2R-Fc proteins and not to irrelevant control Fc fusion proteins, Fc protein and bovine-serum albumin. Competitive ELISA of Fab-phage binding to human, canine and murine IGF2R (**b**) IGF2R-Fab1; (**c**) IGF2R-Fab2; and (**d**) IGF2R-Fab3, indicating low-nanomolar affinity.

Following phage library panning, we isolated 1 sequence (IGF2R-Fab-1) from the naïve library that repeated multiple times. We had several hits from synthetic libraries but selected two clones (IGF2R-Fab-2, IGF2R-Fab-3) that showed comparable ELISA signals to all IGF2R fragments. Phage ELISA confirmed specific binding of isolated Fab-phage to IGF2R with no binding to control Fc fusion proteins (Figure 2a). Competitive Fab-phage ELISA revealed tight binding to human, murine and canine IGF2R recombinant proteins (Figure 2b–d). While the competitive ELISA presented here is semi-quantitative, these data indicate a similar IC_50_ of binding to human, murine and canine IGF2R in the low-nanomolar range.

### 3.2. Fab’ Fragments Bound to IGF2R and Were Cytocidal to IGF2R Positive Cells In Vitro

**Figure 3:**
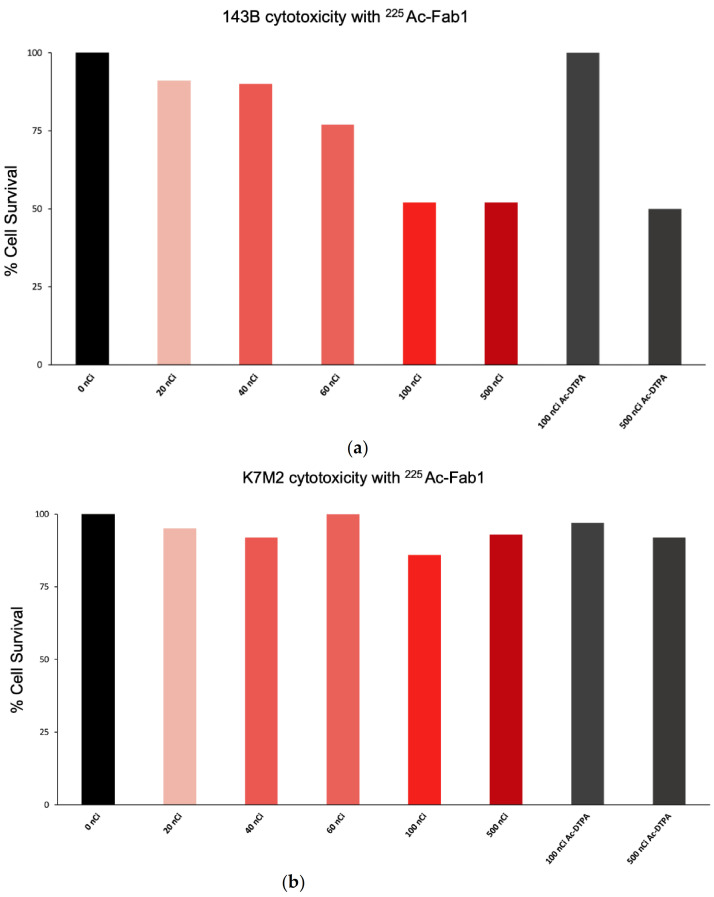
In vitro cytotoxicity of ^225^Ac-labeled Fab1 towards OS cell lines. (**a**) IGF2R-positive human 143B cells; (**b**) IGF2R-negative murine K7M2 OS cells. 100 and 500 nCi of ^225^Ac-DTPA served as controls to demonstrate the antibody-related specificity of cell killing.

Purified Fab proteins, when radiolabeled with the alpha-emitter ^225^Ac- Fab1 killed IGF2R-positive 143B human OS cells in a dose dependent manner (Figure 3a) while no killing of IGF2R-negative K7M2 murine OS cells was observed (Figure 3b). The cell killing was IGF2R and antibody-specific, as no killing of 143B cells with 100 nCi ^225^Ac-DTPA complex was observed, while cell killing with 500 nCi ^225^Ac-DTPA was due to high concentration of radioactivity in the small volume of the sample.

### 3.3. Full Size Human IgGs Bound to IGF2R from Different Species, to Human and Canine OS Patient Derived Cell Lines, and to Canine Tumors from Companion Dogs

**Figure 4:**
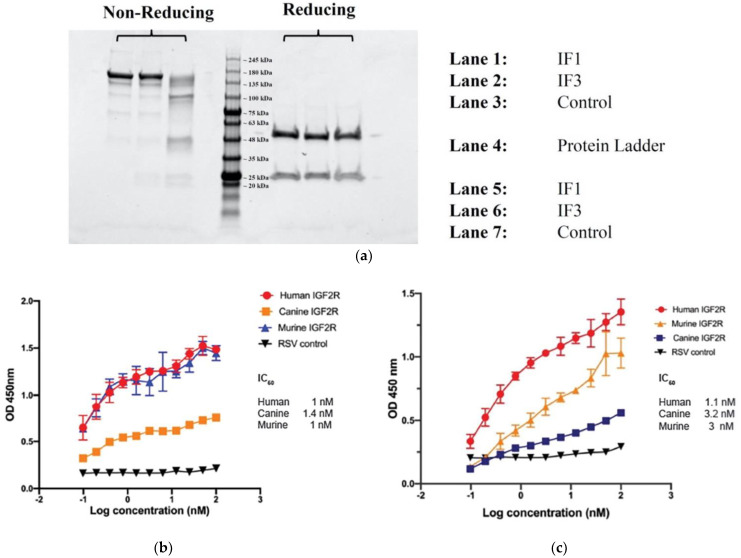
Characterization of purified IgGs IF1 and IF3. (**a**) SDS-PAGE gel analysis of purified proteins. (**b**) Binding of IgG IF1 to recombinant IGF2R-Fc proteins. (**c**) Binding of IgG IF3 to recombinant IGF2R-Fc proteins. IC_50_ values indicated below. Palivizumab (IgG1) against respiratory syncytial virus (RSV) was used as a negative control.

**Figure 5:**
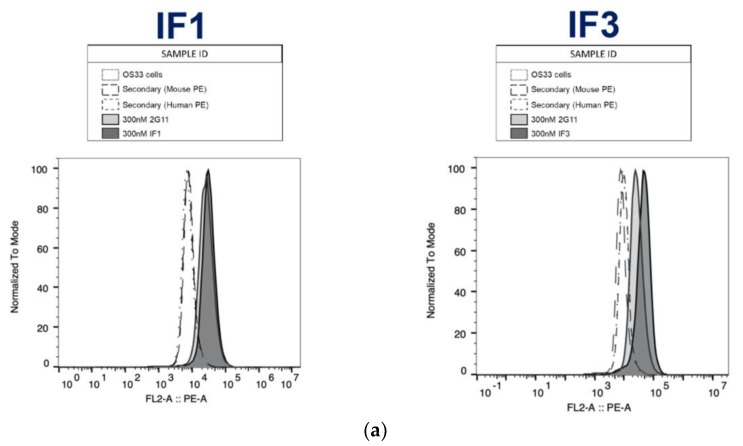

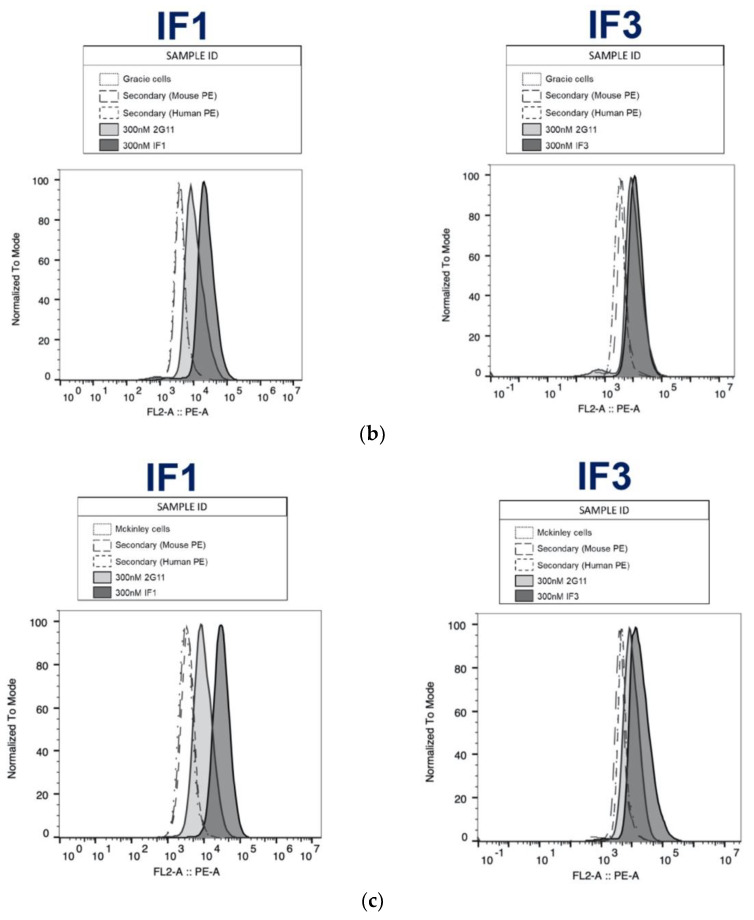
Binding of IF1 and IF3 mAbs to human and canine patient-derived OS cells by flow cytometry. (**a**) OS-33 (human); (**b**) Gracie (canine); (**c**) McKinley (canine). Commercial murine mAb 2G11 that is cross-reactive to human and canine IGF2R was used as a positive control.

**Figure 6:**
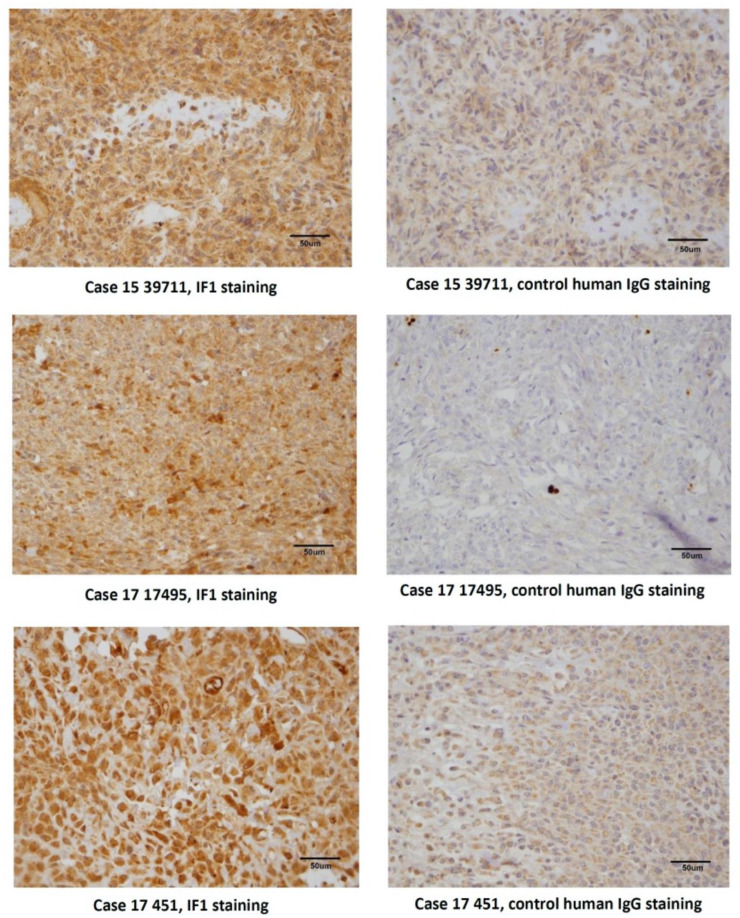
Immunohistochemistry of IF1 binding to randomly selected canine OS tumors. Human antibody palivizumab to respiratory syncytial virus (RSV) was used as a negative control (right panels). Original magnification 400×, size bar 50 μm.

IGF2R-Fab1, 2 and 3 were converted to full-length human IgGs and expressed in human origin Expi293F cells. IgG versions of Fab1 and Fab3 (referred further in the text as IF1 and IF3) expressed well but Fab2 resulted in very low yield. Purified proteins were characterized using SDS-PAGE (Figure 4a) and demonstrated specific binding to human, canine and murine IGF2R by ELISA with nanomolar affinity (Figure 4b,c). Flow cytometry showed that IF1 and IF3 specifically bound to patients derived cell lines from human and canine patients (Figure 5). Figure 6 shows IHC of IF1 binding to randomly selected tumors from companion dogs with OS while control human mAb palivizumab demonstrated no or very weak binding (Figure 6).

### 3.4. Conjugation of IF1 and IF3 Linker CHXA” Did Not Interfere with Their Immunoreactivity towards IGF2R and Their Structural Integrity

**Figure 7:**
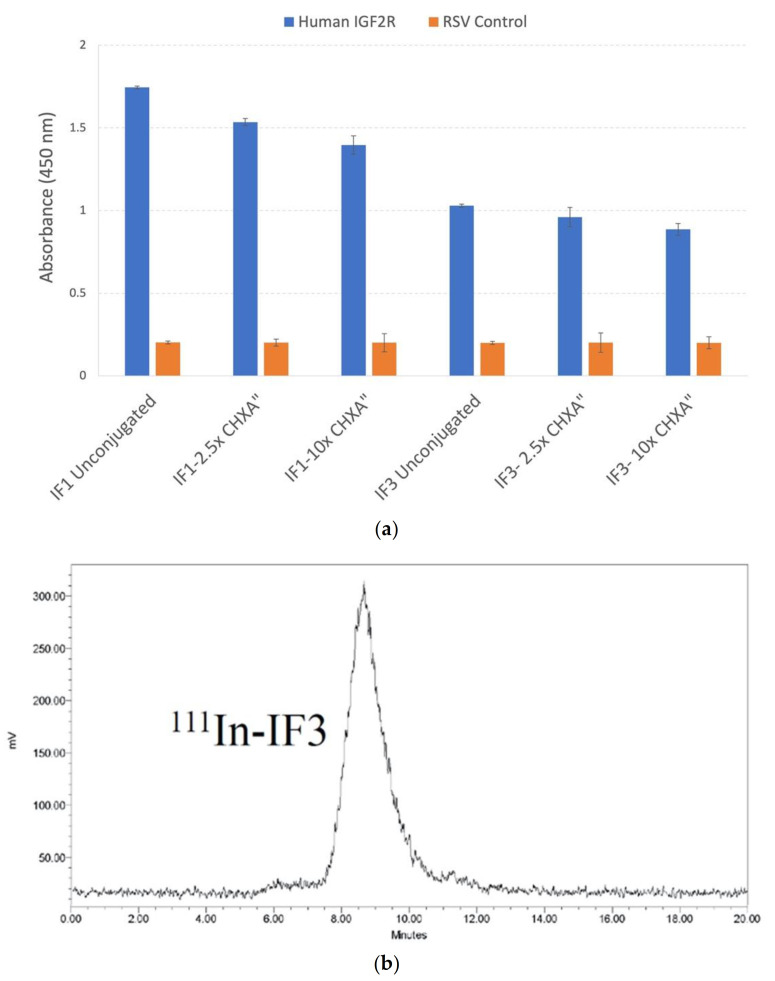
Characterization of IF1 and IF3 conjugated to 2.5-fold and 10-fold excess molar ratio of CHXA” bifunctional linker by IGF2R ELISA and by HPLC: (**a**) IGF2R ELISA. Palivizumab (IgG1) against respiratory syncytial virus (RSV) was used as a negative control; (**b**) size exclusion HPLC of ^111^In-labeled IF3.

Next, we conjugated IF1 and IF3 with the bifunctional linker CHXA” which enables radiolabeling of the mAbs with diagnostic and therapeutic radionuclides. Comparative ELISA revealed that both antibodies also preserved significant percentage of their immunoreactivity when conjugated to 2.5 and 10 initial molar ratios of CHXA” bifunctional linker to the antibody, with 2.5 molar ratio having lesser effect on the immunoreactivity of the mAbs (Figure 7a). HPLC of ^111^In-labeled antibodies demonstrated a single peak (Figure 7b) attesting to the structural integrity of the radiolabeled antibodies. These data provided impetus for in vivo evaluation of the conjugated mAbs.

### 3.5. IF1 and IF3 Showed Different Clearance from the Blood

**Figure 8:**
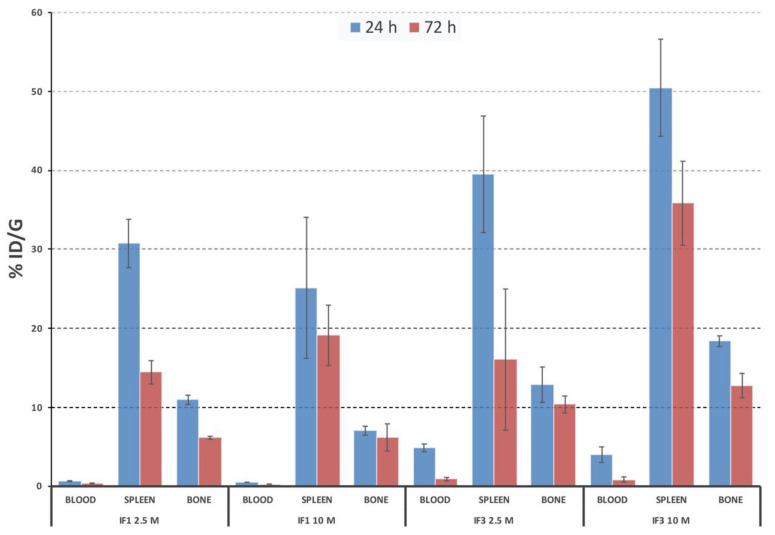
Biodistribution of ^111^In-labeled IF1 and IF3 in female SCID mice at 24 and 72 h post antibodies administration. 2.5 and 10 initial molar ratios of CHXA” bifunctional linker to the antibody were used.

IF1 and IF3 mAbs conjugated with 2.5 and 10 excess molar ratios of CHXA” were radiolabeled with ^111^In and a pilot biodistribution was performed in healthy female SCID mice at 24 and 72 h post mAb administration to evaluate mAb uptake and clearance from the blood, spleen and bone. Slower blood clearance of IF3 was observed in comparison with IF1—where at 24 h post injection time-point there was only 0.7% ID/g of IF1 in the blood compared to 4.7% ID/g of IF3. Both antibodies showed considerable uptake in the spleen and some uptake in the bone, which decreased from 24 to 72 h. The higher amount of CHXA” ligand attached to either antibody slightly increased the clearance from the blood (Figure 8). Given that fast blood clearance might not be suitable for imaging and therapy, we selected IF3 conjugated to 2.5 initial CHXA” to mAb molar ratio for further experiments.

### 3.6. IF3 localized in Human and Canine OS Tumors in Mice

**Figure 9:**
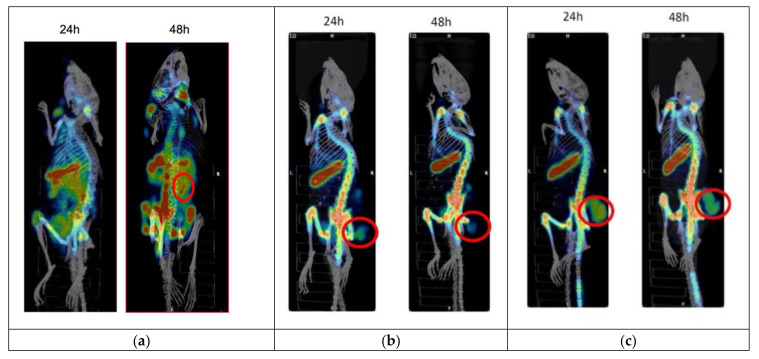
microSPECT/CT imaging of human and canine OS bearing mice. Female SCID mice were implanted with 143B human cells and OS33 patient derived osteosarcoma cells and with canine patient-derived Gracie cells imaged with ^111^In-labeled IF3 at 24 or 48 h post antibody administration. (**a**) Mouse with 143B tumor at 24 and 48 h; (**b**) mouse with OS-33 tumor at 24 and 48 h; (**c**) mouse with Gracie tumor at 24 and 48 h. The tumor uptake of the radiolabeled antibody on the images is shown with red circles. Red color indicates the highest accumulation of activity in tissue, blue—the lowest.

^111^In-labeled IF3 was administered to female mice bearing 143B human OS tumors, human patient derived OS33 tumors, or canine patient derived Gracie tumors. Figure 9 shows clearly visible tumor uptake at 24 or 48 h for all three models (Figure 9). Significant uptake in the spleen was also observed.

## 4. Discussion

Here, we describe the creation and preliminary evaluation of human antibodies to IGF2R as potential agents for radioimmunoimaging and RIT of OS in children, adolescents and companion animals. We have started with identifying the region on IGF2R that can be expressed and purified readily and is highly conserved among humans, canines and mice. The extracellular domain of IGF2R contains 15 cation-independent mannose receptor (CIMR) domains repeats and one FNII domain located between CIMR domains 12 and 13. IGF2R contains binding sites for IGFII and phospho-mannosyl moieties. Mannosylated proteins bind domains 3, 5 and 9 [23]. IGFII binding site is in domain 11, with domain 13 and FNII domains assisting the binding of IGFII to IGF2R [24]. Human, mouse and canine IGF2R genes comprising of the IGFII binding region (containing domains 11–13) were sequence aligned and shows that this region is highly conserved across these species with 82% sequence identity (Figure S1). Human IGF2R domains 11–13 were previously recombinantly expressed for structural studies [24]. We therefore expressed homologous sequences from murine and canine IGF2R in addition to human IGF2R as Fc fusion proteins. This conserved region enabled generation of the antibodies that are cross-reactive.

To generate human antibodies that bind to conserved regions of IGF2R, we developed both synthetic and naïve antibody Fab-fragment libraries. While elaborate and sophisticated fully human naïve and synthetic antibody phage-display libraries have been described before (see Reference [25] for a recent review), we used a simple cost-effective method to generate preliminary libraries for this project. We used trastuzumab 4D5-8 clone as a template and used structure guided mutagenesis to generate our synthetic library (Figure 1). We used a conservative approach to mutate only solvent exposed amino acids in CDRL1, CDRL2, CDRH1 and CDRH2. Solvent exposed regions of CDRL3 and CDRH3 were further mutated to vary the loop lengths to generate additional diversity. To make cost-effective libraries, we used a custom codon (N1)HT that only encodes for 12 amino acids, while generating a bias for Tyr and Ser residues (Figure 1 and Supplementary Materials. This approach eliminates the need for expensive mutagenic oligonucleotides. We generated a library of 5 × 10^9^ variants. For the naïve library, we used primers to amplify antibody repertoire from pooled peripheral blood monocyte cDNA. We focused on variable heavy chain genes from the VH3 family and variable light chain genes from the kappa family, as these are the most abundant in immune repertoires. These genes were cloned into display vector pHP153 to generate a naïve library of 3 × 10^8^ variants. Following phage panning of both libraries, we successfully isolated three Fabs that were cross-reactive to human, murine and canine IGF2R with high specificity and affinity.

The lead antibodies were expressed as full-length IgGs in human suspension cell line Expi293F. Cross-reactivity of the antibodies was confirmed by their binding to human derived tumors in vivo, to mouse spleens and canine spontaneous tumors via IHC. In vivo evaluation of the candidate mAbs IF1 and IF3 also revealed very fast clearance of ^111^In-labeled IF1 from the blood. While fast clearance from the blood can potentially assist with imaging by improving target to non-target ratio and potentially minimizing toxicity to the bone marrow—excessively fast clearance might prevent the mAb to achieve significant uptake in the tumor to deliver the cytocidal dose of radiation to the tumor cells when RIT is attempted. For that reason, IF3 with its slower clearance from the blood was selected for evaluation in human and canine patients derived tumor models. ^111^In-labeled IF3 demonstrated uptake in both human and canine patient derived tumors in SCID mice (Figure 9). In addition, minimizing the number of CHXA” linker molecules attached to the antibodies also helped to ensure that the antibodies stay in circulation for a longer time. A possible explanation of this observation could be that excessive negative charge of the antibody decorated with several CHXA” molecules contributes to its faster clearance from the blood.

## 5. Conclusions

Several novel strategies have been tried for therapy of OS but, so far, only immunotherapy with unlabeled mAbs has been evaluated in clinical trials [26]. In regard to TRT for OS, in some preclinical studies, ^153^Samarium-EDTMP or polymeric radiolabeled phosphonates were suggested for targeted therapy of OS [15,27]. Larsen et al. performed in vitro evaluation of ^211^Astatine-labeled TP3 mAb for killing of OS cells [9]. Very recently, the same group armed anti-CD146 mAb with 177Lutetium and reported biodistribution and dosimetry results in a mouse model of OS [28]. Clearly, the novel human pan-antibodies binding to human, canine and murine IGF2R are promising reagents for further evaluation and development for radioimmunoimaging and RIT of OS in human and canine patients. Given the lack of considerable progress in developing new treatments for the metastatic form of this disease in the last 20 years, further development of RIT as a treatment option for OS is much needed.

## 6. Patents

A provisional patent application titled “Antibodies To IGF2R and Methods” has been filed with E.D. and M.U. as inventors.

## Data Availability

All data presented in this study are available in this article and Supplementary Material.

## Supplementary Materials

The following are available online at https://www.mdpi.com/article/10.3390/cancers13092208/s1; Figure S1: Sequence alignment of IGF2 binding domains 11–13 of IGF2R from different species, Table S1: Primary PCR primers, Table S2: Secondary PCR primers. Additional information on materials and methods for construction of human antibody phage-display.
