# Supplementary material for: Novel Human Antibodies to Insulin Growth Factor 2 Receptor (IGF2R) for Radioimmunoimaging and Therapy of Canine and Human Osteosarcoma
Source: Cancers (Basel). 2021 May 4;13(9):2208. doi: 10.3390/cancers13092208 (PMC8124616; doi:10.3390/cancers13092208)
Supplement: Supplementary file 1 [file cancers-13-02208-s001.zip › cancers-1190938-supplementary.pdf]

Supplementary Materials:

1. Synthetic library construction: Materials and Methods

1.1 4D5-8 Clone sequence cloned into pHP153 plasmid:

VL

```
gatatccagatgacccagtccccgagctccctgtccgcctctgtgggcgatagggtcacc
 D  I  Q  M  T  Q  S  P  S  S  L  S  A  S  V  G  D  R  V  T

atcacctgccgtgccagtcagGATGTGAATACCgctgtagcctggtatcaacagaaacca
 I  T  C  R  A  S  Q  D  V  N  T  A  V  A  W  Y  Q  Q  K  P

ggaaaagctccgaagcttctgatttactcggcatccTTCctctactctggagtcccttct
 G  K  A  P  K  L  L  I  Y  S  A  S  F  L  Y  S  G  V  P  S

cgcttctctggtagccgttccgggacggatttcactctgaccatcagcagtctgcagccg
 R  F  S  G  S  R  S  G  T  D  F  T  L  T  I  S  S  L  Q  P

gaagacttcgcaacttattactgtcagcaaCACTACACCACCCCACCAacgttcggacag
 E  D  F  A  T  Y  Y  C  Q  Q  H  Y  T  T  P  P  T  F  G  Q

ggtaccaaggtggagatcaaa
 G  T  K  V  E  I  K
```

VH

```
gaggttcagctggtggagtctggcggtggcctggtgcagccaggggggctcactccgtttg
 E  V  Q  L  V  E  S  G  G  G  L  V  Q  P  G  G  S  L  R  L

tcctgtgcagcttctggcttcaacattaaggacacctacattcactgggtgcgtcaggcc
 S  C  A  A  S  G  F  N  I  K  D  T  Y  I  H  W  V  R  Q  A

ccgggtaagggcctggaatgggttgcacgcatctacccaaccaatggctacacccgctat
 P  G  K  G  L  E  W  V  A  R  I  Y  P  T  N  G  Y  T  R  Y

gccgatagcgtcaagggccgtttcactataagcgcagacacatccaaaaacacagcctac
 A  D  S  V  K  G  R  F  T  I  S  A  D  T  S  K  N  T  A  Y

ctacaaatgaacagcttaagagctgaggacactgccgtctattattgtagccgctggggc
 L  Q  M  N  S  L  R  A  E  D  T  A  V  Y  Y  C  S  R  W  G

ggcgacggctttacgccatggactactggggtcaaggaaccctggtcaccgtctcctcg
 G  D  G  F  Y  A  M  D  Y  W  G  Q  G  T  L  V  T  V  S  S
```

1.2 Mutagenic Oligonucleotides:

The 4D5-8 synthetic library was constructed by site-directed mutagenesis using the following mutagenic oligonucleotides ordered from Integrated DNA Technologies (IDT). These oligonucleotides utilize a hand-mixed base N1 which consists of 10%A, 20%C, 25%G, 45%T.

4D5-Lib1-L1
CCTGCCGTGCCAGTCAG (N1:10202545)HT GTG (N1)HT ACCGCTGTAGCCTGGTATC

4D5-Lib1-L2
CGAAGCTTCTGATTTAC(N1:10202545)HT GCATCC (N1)HT CTCTACTCTGGAGTCC

4D5-Lib1-L3-4aa
CTTATTACTGTCAGCAA(N1:10202545)HT (N1)HT (N1)HT (N1)HT
CCACCAACGTTCGGAC

4D5-Lib1-L3-5aa
CTTATTACTGTCAGCAA(N1:10202545)HT (N1)HT (N1)HT (N1)HT (N1)HT
CCACCAACGTTCGGAC

4D5-Lib1-L3-6aa
CTTATTACTGTCAGCAA(N1:10202545)HT (N1)HT (N1)HT (N1)HT (N1)HT (N1)HT
CCACCAACGTTCGGAC

4D5-Lib1-L3-7aa
CTTATTACTGTCAGCAA(N1:10202545)HT (N1)HT (N1)HT (N1)HT (N1)HT (N1)HT
(N1)HT CCACCAACGTTCGGAC

4D5-Lib1-H1
GGCTTCAACATTAAG(N1:10202545)HT ACC (N1)HT ATTCACTGGGTGCGTC

4D5-Lib1-H2
CTGGAATGGGTTGCA (N1:10202545)HT ATC (N1)HT CCA (N1)HT (N1)HT GGC (N1)HT
ACCCGCTATGCCGATAG

4D5-Lib1-H3-4aa
CTATTATTGTAGCCGCTGG (N1:10202545)HT (N1)HT (N1)HT (N1)HT
GCCATGGACTACTGG

4D5-Lib1-H3-5aa
CTATTATTGTAGCCGCTGG (N1:10202545)HT (N1)HT (N1)HT (N1)HT (N1)HT
GCCATGGACTACTGG

4D5-Lib1-H3-6aa
CTATTATTGTAGCCGCTGG (N1:10202545)HT (N1)HT (N1)HT (N1)HT (N1)HT (N1)HT
GCCATGGACTACTGG

4D5-Lib1-H3-7aa
CTATTATTGTAGCCGCTGG (N1:10202545)HT (N1)HT (N1)HT (N1)HT (N1)HT (N1)HT
(N1)HT GCCATGGACTACTGG

4D5-Lib1-H3-8aa

CTATTATTGTAGCCGCTGG (N1:10202545)HT (N1)HT (N1)HT (N1)HT (N1)HT (N1)HT (N1)HT (N1)HT GCCATGGACTACTGG

4D5-Lib1-H3-9aa
CTATTATTGTAGCCGCTGG (N1:10202545)HT (N1)HT (N1)HT (N1)HT (N1)HT (N1)HT (N1)HT (N1)HT (N1)HT GCCATGGACTACTGG

4D5-Lib1-H3-10aa
CTATTATTGTAGCCGCTGG (N1:10202545)HT (N1)HT (N1)HT (N1)HT (N1)HT (N1)HT (N1)HT (N1)HT (N1)HT (N1)HT GCCATGGACTACTGG

4D5-Lib1-H3-11aa
CTATTATTGTAGCCGCTGG (N1:10202545)HT (N1)HT (N1)HT (N1)HT (N1)HT (N1)HT (N1)HT (N1)HT (N1)HT (N1)HT (N1)HT GCCATGGACTACTGG

1.3 Library Construction Methodology: Site-directed mutagenesis was performed as described previously[1] except that all six CDRs were mutated in the same reaction. Oligonucleotides for CDRL3 and CDRH3 were pooled together as they anneal to same region. Given the high number of oligonucleotides for CDRH3 randomization, the reaction was split into two mixes (Set1: 4D5-Lib1-H3-4aa to 4D5-Lib1-H3-7aa; Set2: 4D5). Two 10µg scale reactions were conducted with each set of CDRH3 mixes. The covalently coupled double-stranded DNA from both reactions was then pooled together and electroporated into *E.Coli.* SS320 that were pre-infected with helper phage. We obtained ~3.3 x 10^9 transformants.

2. Naïve Library Construction

We cloned the antibody repertoire from pooled human peripheral leukocyte poly A+ RNA (Takara Bio). We used Thermoscientific Maxima H Minus First strand cDNA synthesis kit to generate single-strand cDNA template from 500ng poly A+ RNA using kit instructions. We used the following set of primers to generate primary PCR products corresponding to VH and VL repertoire.

Table S1: Primary PCR primers

MUBV_VK1a	GMCATCCAGATGACCCAGTCTCCATCC	Forward
MUBV_VK1b	GMCATCCAGTTGACCCAGTCTCCATCC	Forward
MUBV_VK1c	GCCATCCGGATGACCCAGTCTCCATYC	Forward
MUBV_Vk1d	GACATCCAGATGACCCAGTCTCCATCTTC	Forward
MUBV_VK1e	GACATCCAGATGACCCAGTCTCCWTCC	Forward
MUBV_VK3a	GAAATTGTGTTGACGCAGTCTCCAGSCACC	Forward
MUBV_VK3b	GAAATAGTGATGACGCAGTCTCCAGCCACC	Forward
MUBV_VK3c	GAAATTGTGTTGACACAGTCTCCAGCC	Forward
MUBV_CHK	ACACTCTCCCCTGTTGAAGCTCTT	Reverse
MUBV_VH3_1L	GAGGTGCAGCTGGTGGAGTCTGGGGGAGGCTTGGTCCAG	Forward

MUBV_VH3_2L	CAGGTGCAGCTGGTGGAGTCTGGGGGAGGCTTGGTCAAG	Forward
MUBV_VH3_3L	GAGGTGCAGCTGGTGGAGTCTGGGGGAGGCTTGGTAAAG	Forward
MUBV_VH3_4L	GAGGTGCAGCTGGTGGAGTCTGGGGGAGGTGTGGTACGG	Forward
MUBV_VH3_5L	CAGGTGCAGCTGGTGGAGTCTGGGGGAGGCGTGGTCCAG	Forward
MUBV_VH3_6L	GAGGTGCAGCTGGTGGAGACTGGAGGAGGCTTGATCCAG	Forward
MUBV_JH1_2	TGAGGAGACRGTGACCAGGGTG	Reverse
MUBV_JH3	TGAAGAGACGGTGACCATTGT	Reverse
MUBV_JH4_5	TGAGGAGACGGTGACCAGGGTT	Reverse
MUBV_JH6	TGAGGAGACGGTGACCGTGGTCC	Reverse

200µl scale PCR reactions were setup using Phusion DNA polymerase using manufacturer recommended protocols for each of VK1, VK3 and VH3. Forward and Reverse primers for each subfamily as listed in the table were pooled together. 2.5µl of first-strand cDNA was used as a template and a gradient annealing temperature of 63-72°C was used for each PCR reaction. Following electrophoresis, bands of ~680bp for VL and ~380bp for VH were excised from the gel and purified using gel extraction kit.

A secondary PCR with overhangs to add restriction sites was setup at the 400µl scale with 200ng of primary PCR product as the template. The following primers were used:

Table S2: Secondary PCR primers

**Primer Name**	**Primer Sequence**	**Primer direction**
MUBV_SEC_VK1a	aaatgcctatgcatcg GMC ATC CAG ATG ACC CAG TCT CCA	Forward
MUBV_SEC_VK1b	aaatgcctatgcatcg GMC ATC CAG TTG ACC CAG TCT CCA	Forward
MUBV_SEC_VK1c	aaatgcctatgcatcg GCC ATC CGG ATG ACC CAG TCT CCA	Forward
MUBV_SEC_Vk1d	aaatgcctatgcatcg GAC ATC CAG ATG ACC CAG TCT CCA	Forward
MUBV_SEC_VK1e	aaatgcctatgcatcg GAC ATC CAG ATG ACC CAG TCT CCW	Forward
MUBV_SEC_VK3a	aaatgcctatgcatcg GAA ATT GTG TTG ACG CAG TCT CCA	Forward
MUBV_SEC_VK3b	aaatgcctatgcatcg GAA ATA GTG ATG ACG CAG TCT CCA	Forward
MUBV_SEC_VK3c	aaatgcctatgcatcg GAA ATT GTG TTG ACA CAG TCT CCA	Forward
SAM_SEC_JK1	**GAAGATGAAGACAGATGGTGCAGCCACCGTACG** TTTGATTTCCACCTTGGTCC	Reverse

SAM_SEC_JK2	**GAAGATGAAGACAGATGGTGCAGCCACCGTACG** TTTGATCTCCAGCTTGGTCC	Reverse
SAM_SEC_JK34	**GAAGATGAAGACAGATGGTGCAGCCACCGTACG** TTTGATMTCCACYTTGGTCC	Reverse
SAM_SEC_JK5	**GAAGATGAAGACAGATGGTGCAGCCACCGTACG** TTTAATCTCCAGTCGTGTCC	Reverse
SAM_SEC_VH3a	**CTTCTTGCATCTATGTTCGTTTTTCTATTGCTACAAACG CGTATGCT**GAGGTGCAGCTGGTGGAG	FORWARD
SAM_SEC_VH3b	**CTTCTTGCATCTATGTTCGTTTTTCTATTGCTACAAACG CGTATGCT**CAGGTGCAGCTGGTGGAG	FORWARD
MUBV_SEC_JH12	**GGCCTTTTGT GCTAGC TGAGGAGACRGTGACCAG**	Reverse
MUBV_SEC_JH3	**GGCCTTTTGT GCTAGC TGAAGAGACGGTGACCAT**	Reverse
MUBV_SEC_JH45	**GGCCTTTTGT GCTAGC TGAGGAGACGGTGACCAG**	Reverse
MUBV_SEC_JH6	**GGCCTTTTGT GCTAGC TGAGGAGACGGTGACCGT**	Reverse

Following secondary PCR, bands of~380 bp for VK1/VK3 and ~400 bp for VH3 were gel-extracted and purified. 200ng of light chain and heavy chain PCR products were spliced with 300ng of dsDNA with following intergenic sequence using Splicing Overlap Extension (SOE) PCR.

Intergenic sequence (encoding CL and internal ribosome entry sites):

```
Cgtacggtggctgcaccatctgtcttcatcttcccgccatctgatgaacagttgaaatctggaactgcct
ctgttgtgtgcctgctgaataacttctatcccagagaggccaaagtacagtggaaggtggataacgccct
ccaatcgggtaactcccaggagagtgtcacagagcaggacagcaaggacagcacctacagcctcagcagc
accctgacgctgagcaaagcagactacgaaaaacataaagtctacgcctgcgaagtcacccatcagggcc
tgagctcgcccgtcacaaagagcttcaacaggggagagtgtggtggttctgattacaaagatgacgatga
caaataattaactcgaggctgagcaaagcagactactaataacataaagtctacgccggacgcatcgtgg
ccctagtacgcaagttcacgtaaaaagggtaactagaggttgaggtgattttatgaaaaagaatatcgca
tttcttcttgcatctatgttcgtttttctattgctacaaacgcgtatgct
```

Following SOE PCR with light-chain forward primer pool and heavy chain reverse primer pool, a band corresponding to the assembled product (~1250 bp) was gel extracted and purified. Vector pHP153 and purified PCR products were digested with restriction enzymes NsiI and NheI and purified using gel-extraction kit. Two ligation reactions (K1 and K3) were setup using 1.5µg vector and 2.5µg insert to give a molar ratio of (1:5) using T4 DNA Ligase at 16°C overnight. The DNA was purified on a QIAquick columns and pooled together for electroporation. Following electroporation ~4 x 10^8 transformants were obtained. Sequencing analysis showed that only 25% of clones had inserts giving a library size of 10^8 variants.

Supplementary Figures

```
CLUSTAL O(1.2.4) multiple sequence alignment

Human   NEHDDCQVTNPSTGHLFDLSSLSGRAGFTAAYSEKGLVYMSICGENENCPPGVGACFGQT 60
Mouse   NTHDDCQVTNPSTGHLFDLSSLSGRAGINASYSEKGLVFMSICEENENCGPGVGACFGQT 60
Canine  NVHDNCQVTNPATGHLFDLSSLSGRAGHTAAYSEKGLVYISICEDNENCSPGVGACFGQT 60
        * **:******:*************** .*:*******::*** :**** **********

Human   RISVGKANKRLRYVDQVLQLVYKDGSPCPSKSGLSYKSVISFVCRPEARPTNRPMLISLD 120
Mouse   RISVGKASKRLSYKDQVLQLVYENGSPCPSLSDLRYKSVISFVCRPEAGPTNRPMLISLD 120
Canine  RISVGKANKRLTYVDQVLQLVYEDGSPCPSKSGLTYKSVISFVCRPEAGPTNRPMLISLD 120
        *******.*** * ********::****** *.* ************* ***********

Human   KQTCTLFFSWHTPLACEQATECSVRNGSSIVDLSPLIHRTGGYEAYDESEDDASDTNPDF 180
Mouse   KQTCTLFFSWHTPLACEQATECTVRNGSSIIDLSPLIHRTGGYEAYDESEDDTSDTTPDF 180
Canine  KQTCTLFFSWHTPLACEQVTECSVRNGSSIIDLSPLIHRTGGYEAYDESEDDTSDTGPDF 180
        ******************.***:*******:*********************:*** ***

Human   YINICQPLNPMHGVPCPAGAAVCKVPIDGPPIDIGRVAGPPILNPIANEIYLNFESSTPC 240
Mouse   YINICQPLNPMHGVPCPAGASVCKVPVDGPPIDIGRVTGPPIFNPVANEVYLNFESSTHC 240
Canine  YINICQPLNPMHGVPCPAGAAVCKVPVDGPPIDIGRVTGPPILNPIANEVYLNFESSTPC 240
        ********************:*****:**********:****:**:***:******** *

Human   LADKHFNYTSLIAFHCKRGVSMGTPKLLRTSECDFVFEWETPVVCPDEVRMDGCTLTDEQ 300
Mouse   LADRYMNYTSLITFHCKRGVSMGTPKLIRTNDCDFVFEWETPIVCPDEVKTQGCAVTDEQ 300
Canine  LADKHFNYTSLIAFHCRRGVSMGAPMLLRTSDCDFVFGWETPLVCPDEVKMDGCSLTDEQ 300
        ***:::******:***:******:* *:**.:***** ****:******: :**::****

Human   LLYSFNLSSLSTSTFKVTRDSRTYSVGVCTFAVGPEQGGCKDGGVCLLSGTKGASFGRLQ 360
Mouse   LLYSFNLTSLSTSTFKVTRDARTYSIGVCTAAAGLGQEGCKDGGVCLLSGNKGASFGRLA 360
Canine  LHYSFNLSSLSKSTFKVTRDSRTYSIGVCTAAAGLDEGGCKDGGVCLLSGSKGASFGRLA 360
        * *****:***.********:****:**** *.*  : *************.********

Human   SMKLDYRHQDEAVVLSYVNGDRCPPETDDGVPCVFPFIFNGKSYEECIIESRAKLWCSTT 420
Mouse   SMQLDYRHQDEAVILSYVNGDPCPPETDDGEPCVFPFIYKGKSYDECVLEGRAKLWCSKT 420
Canine  SMRLDYRHQDEAVILSYANGDNCPPETEAGDPCVFPFIFNGKSYEECVVEGRARLWCSTT 420
        **:**********:***.*** *****: * *******::****:**::*.**:****.*

Human   ADYDRDHEWGFCRHSNSYRTSSIIFKCDEDEDIGRPQVFSEVRGCDVTFEWKTKVVCPP 479
Mouse   ANYDRDHEWGFCRQTNSYRMSAIIFTCDESEDIGRPQVFSEDRGCEVTFEWKTKVVCPP 479
Canine  ANYDRDHEWGFCRHSNSHRMSSIIFKCDEDADIGRPQVFSEVRGCEVTFEWKTKVVCPP 479
        *:***********::**:* *:***.***. ********** ***:*************
```

Figure S1. Sequence alignment of IGF2 binding domains 11-13 of IGF2R from different species

Reference:

1. Fellouse FA SS (2007) Making Antibodies in Bacteria. In: Kaser GCHMR (ed) Making and using antibodies: A practical handbook. CRC Press, Boca Raton, FL, pp 157-180
